# Mutations in the *ATP13A2* Gene and Parkinsonism: A Preliminary Review

**DOI:** 10.1155/2014/371256

**Published:** 2014-08-14

**Authors:** Xinglong Yang, Yanming Xu

**Affiliations:** Department of Neurology, West China Hospital, Sichuan University, 37 Guo Xue Xiang, Chengdu, Sichuan 610041, China

## Abstract

Parkinson's disease (PD) is a major neurodegenerative disorder for which the etiology and pathogenesis remain as elusive as for Alzheimer's disease. PD appears to be caused by genetic and environmental factors, and pedigree and cohort studies have identified numerous susceptibility genes and loci related to PD. Autosomal recessive mutations in the genes *Parkin, Pink1, DJ-1, ATP13A2, PLA2G6*, and *FBXO7* have been linked to PD susceptibility. Such mutations in *ATP13A2*, also named *PARK9*, were first identified in 2006 in a Chilean family and are associated with a juvenile-onset, levodopa-responsive type of Parkinsonism called Kufor-Rakeb syndrome (KRS). KRS involves pyramidal degeneration, supranuclear palsy, and cognitive impairment. Here we review current knowledge about the *ATP13A2* gene, clinical characteristics of patients with PD-associated *ATP13A2* mutations, and models of how the ATP13A2 protein may help prevent neurodegeneration by inhibiting *α*-synuclein aggregation and supporting normal lysosomal and mitochondrial function. We also discuss another *ATP13A2* mutation that is associated with the family of neurodegenerative disorders called neuronal ceroid lipofuscinoses (NCLs), and we propose a single pathway whereby *ATP13A2* mutations may contribute to NCLs and Parkinsonism. Finally, we highlight how studies of mutations in this gene may provide new insights into PD pathogenesis and identify potential therapeutic targets.

## 1. Introduction

Parkinson's disease (PD) is a neurodegenerative disorder for which the etiology and pathogenesis remain elusive, although it is known to be a multifactorial disease involving both genetic and environmental factors. Pedigree and cohort studies of patients with inherited forms of PD, which account for only 5–10% of cases [1], have identified numerous genes and loci associated with PD susceptibility [2, 3]. Autosomal recessive mutations in six of these genes have been linked to the disease:* Parkin* (*PARK2*) [4],* DJ-1* (*PARK7*) [5],* PINK1* (*PARK6*) [6],* ATP13A2* (*PARK9*) [7],* PLA2G6* (*PARK14*) [8], and* FBXO7* (*PARK15*) [9].

Autosomal recessive mutations in the* ATP13A2* gene were first discovered in 2006 in a single Chilean pedigree [7]. Several members of the family showed a rare, juvenile-onset, levodopa-responsive type of Parkinsonism named Kufor-Rakeb syndrome (KRS), involving pyramidal degeneration, supranuclear palsy, and cognitive impairment. Subsequent studies in several other countries linked other mutations to KRS and early-onset Parkinsonism. At the same time,* ATP13A2* mutations have been associated with the occurrence of neurodegenerative disorders called neuronal ceroid lipofuscinoses (NCLs) in patients with Parkinsonism [10]. Some of the NCL-associated mutations overlap with PD-associated ones, suggesting a common pathway in the two types of neurological disease.

Here, we review recent advances in the emerging association of* ATP13A2* mutations with Parkinsonism and NCLs. These findings point to the gene and/or protein as a potential therapeutic target.

## 2. *ATP13A2* Mutations and PD

In the first study linking* ATP13A2* mutations to PD, pedigree analysis of one Chilean family with several members with KRS led to the identification of two loss-of-function mutations: c.1306+5G>A in exon 13 and 3057delC/1019GfsX1021 in exon 26 [7]. In the same study, the authors also performed pedigree analysis of a Jordanian family with several members with KRS, leading to the identification of a 22-bp duplication in exon 16 (1632_1653dup22 or 552LfsX788). This duplication causes a frameshift, resulting in 236 extraneous amino acids followed by a stop codon. All these mutations were absent in a control group of 480 healthy individuals.

Sequencing the complete* ATP13A2* coding region of 46 patients with juvenile- or young-onset PD led to the identification of three additional disease-associated mutations [11]: c.1510G>C/p.Gly504Arg in a Brazilian patient with sporadic PD, c.35C>T/p.Thr12Met (exon 2) in an Italian patient, and c.1597G>A/p.Gly533Arg (exon 16) in another Italian patient. This was the first study to identify any mutation associated with sporadic early-onset PD [11]. Subsequent studies in several countries identified additional novel* ATP13A2* mutations in patients with early-onset disease (Table 1), including studies on individuals from Japan [12, 13], China [14–17], Europe [18], Iran [18, 19], Pakistan [20], Afghanistan [21], Lithuania [22], Inuit communities in Greenland [23], and Italy [24].

The* ATP13A2* mutation c.2236G>A/p.Ala746Thr (exon 20) was identified in three ethnic Chinese individuals from Taiwan and Singapore, two of whom had late-onset PD [14].

However, two subsequent studies failed to detect this mutation in patients with early- or late-onset PD from mainland China and Hong Kong [25–27]. A third study of 65 Chinese patients with early-onset PD detected the Ala746Thr mutation in two patients and four healthy controls [15]. The same study also discovered a novel mutation associated with early-onset disease (c.3274 A>G, Gly1014Ser, exon 26). These studies highlight the need for more research, particularly on Chinese individuals, to identify additional mutations associated with disease and to resolve conflicting results about the Ala746Thr mutation.

Studies using multiplex ligation-dependent probe amplification (MLPA) to measure exon dosage in Iranian patients found deletion of* ATP13A2* exon 2 to be associated with KRS [28]. Three of the 232 affected individuals in the study came from the same family and showed an average age of disease onset of 12 years. Genomic rearrangements were not detected among patients with sporadic or familial PD. In fact, several studies have failed to identify associations of* ATP13A2* mutations with sporadic PD or non-KRS familial PD [25, 29] or with late-onset PD [30]. These findings highlight the need to examine* ATP13A2* mutations in patients with sporadic or familial PD from a broad range of ethnicities, in order to clarify whether the mutations are associated only with juvenile- or young-onset Parkinsonism or perhaps only with KRS.

## 3. Clinical Characteristics of PD Patients Carrying* ATP13A2* Mutations

KRS was initially described in a family with Parkinsonism in the Kufor-Rakeb district in Jordan; affected individuals show a juvenile-onset, levodopa-responsive form of PD involving pyramidal signs, dementia, and supranuclear gaze palsy [31]. These symptoms are quite similar to those of pallidopyramidal syndrome, though KRS differs in that it involves dystonia, which is attributable to pyramidal dysfunction, as well as cognitive dysfunction and supranuclear upgaze paresis [31, 32].

From the literature, we extracted general clinical characteristics of 34 PD patients with* ATP13A2* mutations (Table 2). Most patients had KRS or early-onset disease, either sporadic or familial; two patients had late-onset PD. Slightly more patients were male (21, 56.7%) than female (16, 43.3%); the average age of onset was 23.7 ± 13.8 years. The youngest patient was a 12-year-old Lithuanian boy who had had the disease for 6 years before his case was published; the oldest patient was a 63-year-old Taiwanese woman. Initial symptoms were diverse and included bradykinesia, dystonia, gait disturbance, mental retardation, anxiety, postural instability, and rest tremor. Clinical symptoms were varied and followed the following distribution from the most to the least frequent: rigidity (*n* = 37), bradykinesia (33), postural instability (29), supranuclear upgaze paresis (22), cognitive impairment (19), dystonia (17), resting tremor (17), hallucination (16), and myoclonus (16). A uni- or bilateral Babinski sign was present in 27 of 37 patients.

Most patients were examined by computed tomography (CT) or magnetic resonance imaging (MRI); the most frequent significant features were an enlarged subarachnoid space and diffuse atrophy ranging from mild to severe. Only two patients, an adolescent from Pakistan [20] and an adolescent from Chile [33], showed abnormal bilateral hypointensity in the putaminal and caudate nuclei on T2∗ diffuse MRI images. The clinicians attending the Pakistani patient were able to exclude manganese deposition as the cause of hypointensity, since the patient did not experience manganese exposure or show chronic liver failure; copper deposition, since the patient showed normal serum levels of copper and ceruloplasmin, and the slit lamp test showed no K-F ring; and calcium deposition, since the patient showed normal CT results. In the end, the clinicians attributed the abnormal MRI hypointensity to iron deposition. The clinicians attending the Chilean patient also attributed the hypointensity to ferritin deposits based on the absence of hypointensity on brain CT images, though they did not perform tests to exclude the possibility of deposition of other metals [33].

By single-photon emission CT (SPECT), patient NAPO6, an Italian with* ATP13A2* mutation c.G2629A, showed specific-to-nondisplaceable V′′3 binding ratios that were 75% lower in the caudate and 85% lower in the putamen than those of healthy individuals [24]. His younger brother, designated NAPO7, carried the same* ATP13A2* mutation and showed mild mental retardation but no clinically obvious Parkinsonism. His V′′3 ratio was 40% lower than normal in the caudate and 65% lower in the putamen, consistent with the fact that mild retardation can be an initial symptom of PD [9, 32]. These results suggest that combining genotyping of PD susceptibility genes with positron emission tomography or SPECT may improve diagnosis of early-stage PD, especially in subclinical patients.

## 4. Physiological Role of ATP13A2 and Link to PD

### 4.1. *ATP13A2 *and Function of Lysosomes and Mitochondria

*ATP13A2* encodes a lysosomal transmembrane protein belonging to the 5P-type ATPase subfamily [34]. Wild-type ATP13A2 localizes to the lysosome, while all mutant forms associated with PD localize to the endoplasmic reticulum (ER) [9, 16, 35, 36]. In contrast to genes for other 5P-type ATPases, ATP13A2 in mice is expressed mainly in the brain, suggesting a brain-specific function. ATP13A2 levels in the substantia nigra are substantially lower in postmortem tissue biopsies of patients with sporadic PD than in the corresponding samples from healthy controls [37, 38], but they are higher in survival dopaminergic (DA) neurons of patients than in those of controls [37]. ATP13A2 levels are particularly high in the cytosol of nigral dopaminergic neurons, where the protein accumulates in Lewy bodies [37].

**Figure 1:**
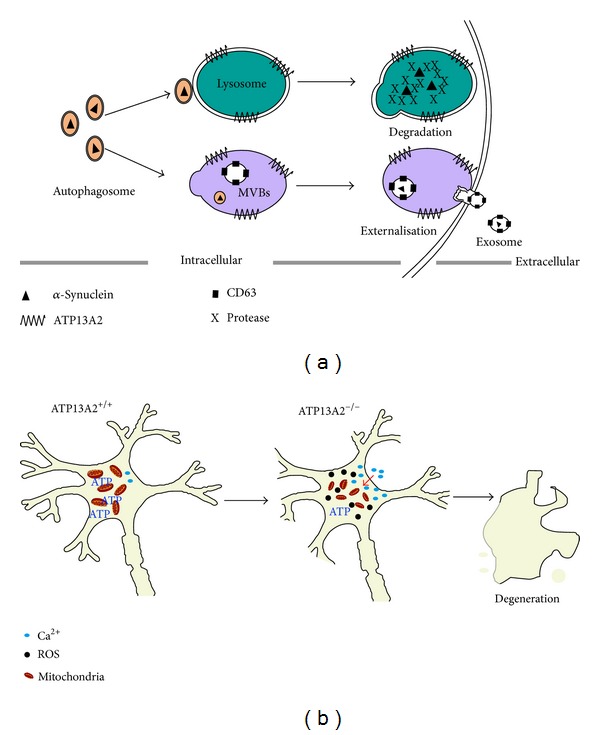
Model of how* ATP13A2* expression may affect lysosomes and mitochondria to prevent neurodegeneration. (a) After *α*-synuclein has been internalized by autophagosomes, it can be immediately degraded in lysosomes containing ATP13A2 or secreted out of the cell via multivesicular bodies (MVBs) also containing ATP13A2. Both routes prevent intracellular accumulation of *α*-synuclein. (b) Knocking out ATP13A2 expression in neurons leads to mitochondrial defects, resulting in higher intracellular levels of reactive oxygen species (ROS) and Ca^2+^, both of which contribute to neurodegeneration.

These circumstantial data implicate ATP13A2 in the pathogenesis and/or progression of PD, but more direct evidence requires insights into the function of the ATP13A2 protein. Studies with cultures of fibroblast cells and DA cells taken from PD patients with* ATP13A2* mutations showed that inhibiting ATP13A2 function decreased the ability of lysosomes to degrade proteins and mediate clearance of autophagosomes [37]. These cellular functions returned to near-normal levels after ATP13A2 activity was restored. These results suggest that ATP13A2 is required for normal lysosome function, which is in turn required for preventing *α*-synuclein aggregation in neurons (Figure 1(a)). This aggregation is a pathological hallmark of both sporadic and familial PD [39].

Several additional studies provide further evidence that ATP13A2 prevents *α*-synuclein aggregation. SH-SY5Y cultures overexpressing ATP13A2 showed lower intracellular levels of *α*-synuclein, perhaps because of increased *α*-synuclein export via multivesicular bodies (MVBs) (Figure 1(a)) [40]. In both whole-animal and neuronal culture models of PD, coexpressing ATP13A2 with *α*-synuclein led to lower synuclein levels in DA neurons than expressing synuclein alone [41]. Neuronal cultures lacking the* ATP13A2* gene showed significantly higher endogenous levels of *α*-synuclein than did the corresponding wild-type neurons [42]. Intriguingly the* ATP13A2*-knockout neurons did not show elevated levels or aggregates of tau protein, which may play an important role in the pathogenesis of Alzheimer's disease (AD). This raises the possibility that ATP13A2 interacts preferentially with *α*-synuclein, consistent with a recent study showing that ATP13A2 colocalized with *α*-synuclein in Lewy bodies but not with *β*-amyloid [38].

In addition to ensuring proper lysosomal function, ATP13A2 may work in mitochondria, such that the reduced activity of* ATP13A2* mutants may lead to mitochondrial defects that contribute to neurodegeneration (Figure 1(b)) [43]. Fibroblasts from patients with KRS showed lower mitochondrial membrane potential and ATP synthesis rates than fibroblasts from healthy individuals [33]. Cell cultures deficient in ATP13A2 showed lower levels of autophagy than healthy cells, leading to higher levels of reactive oxygen species and concomitant oxidative stress [44]. Overexpressing ATP13A2 in neurons inhibited cadmium-induced mitochondrial fragmentation, while silencing ATP13A2 expression induced mitochondrial fragmentation [45] (Figure 1(b)). That same study further showed that increasing or decreasing ATP13A2 expression substantially shortened the neurites of primary midbrain DA neurons, without affecting neurites of cortical neurons. This may mean that the morphological and functional integrity of DA neurons depends on well-controlled ATP13A2 expression [45].

The available evidence suggests that ATP13A2, by supporting lysosomal and mitochondrial function, helps prevent the *α*-synuclein aggregation associated with Parkinsonism [46–49]. The implication is that the* ATP13A2* mutations linked to KRS and other forms of PD are loss-of-function mutations that reduce ATP13A2 activity sufficiently to induce neurodegeneration. Future studies should examine in detail the activity, localization, and binding partners of these mutant proteins.

### 4.2. ATP13A2 and Cation Accumulation

ATP13A2 plays a critical role in the transmembrane transport of manganese and zinc and perhaps of iron and cadmium as well [15]; abnormal accumulation of any of these cations can cause neurodegeneration [41, 50–52]. Thus, patients with PD have been reported to show elevated levels of manganese and zinc in serum and cerebrospinal fluid [53–55], and manganese and zinc exposure are significant environmental risk factors for PD [56, 57]. ATP13A2 helps protect cells from this toxicity by regulating the homeostasis of manganese and zinc in neurons [41, 44, 58, 59]. It may be that dysregulation of ATP13A2 expression disrupts the homeostasis of manganese and zinc in the brain, leading to neurodegeneration.

This possibility is consistent with the interpretation of the abnormal hypointensity in the putamina and caudate nuclei of patients with KRS in T2∗ diffuse MRI images as iron deposits (see Section 3). This finding led those authors to propose KRS with iron deposits as a distinct condition called neurodegeneration with brain iron accumulation (NBIA) [20]. Indeed, iron accumulation was reported in the substantia nigra of PD patients [60], where it was particularly abundant in DA neurons [61]. Administering the iron chelator deferiprone to an animal model of PD induced by oxidative stress improved motor function and increased dopamine levels in the striatum [62]. In a pilot randomized clinical trial, double-blind and placebo-controlled, deferiprone showed some ability to delay or reverse the progression of PD [62].

How mutations in* ATP13A2* may affect cation deposition is unclear. We speculate that loss-of-function mutations in* ATP13A2* may work similarly to silencing of the* PANK2* gene, which disrupts normal cation transfer and leads to mitochondrial and lysosomal dysfunction and ultimately to cation accumulation in the brain [63, 64]. In this way,* ATP13A2* mutants may trigger deposition of the cations zinc, manganese, and iron, leading to metal-induced oxidative damage and ultimately causing decreases in glutathione peroxidase activity, glutathione (GSH) levels, and mitochondrial Complex I activity, as well as increases in levels of basal lipid peroxidation, free radicals, and glutamate [65–67]. The net result is significant neuronal loss that is the distinguishing pathological feature of PD.

This proposed mechanism implies that regulating or restoring the homeostasis of neurotoxic cations may be a neuroprotective therapy for patients with PD. However, only two of the 37 PD patients with* ATP13A2* mutations that we reviewed showed cation accumulation on T2∗ diffuse MRI images (Table 2), and direct postmortem pathological evidence for metal accumulation in PD is lacking [20, 33]. Further studies are urgently needed to clarify whether* ATP13A2* mutations contribute to PD by increasing susceptibility to cation toxicity.

## 5. *ATP13A2* Mutations: A Link between Parkinsonism and NCLs

*ATP13A2* mutations have been identified not only in patients with Parkinsonism, but also in patients with neuronal ceroid lipofuscinoses (NCLs) [10]. NCLs are a group of neurodegenerative disorders that are also lysosomal storage diseases. Clinical manifestations are seizures, progressive cognitive and motor decline, and failing vision. The pathological hallmark of NCLs is accumulation of autofluorescent lipopigment within neuronal lysosomes [68].

Recently, the mutation c.2429C>G in exon 22 of* ATP13A2,* predicted to result in the amino acid substitution p.Met810Arg, was identified in a Belgian family with NCLs [10]. Affected individuals showed not only typical NCL symptoms but also extrapyramidal involvement. Postmortem pathological examination revealed extensive lipofuscin deposits in the cortex, basal nuclei, cerebellum, and retina—but not the white matter—and electron microscopy showed whorled lamellar inclusions typical of NCLs [10]. A link between* ATP13A2* mutations and NCL pathogenesis is further supported by studies in animal models [69, 70]. In fact, mice deficient in* ATP13A2* exhibited neuronal ceroid lipofuscinosis, *α*-synuclein accumulation, and age-dependent sensorimotor deficits, suggesting that PD and NCLs share a pathogenic mechanism [71].

A shared disease pathway may help explain earlier reports of individuals who demonstrate an “overlapping” neurodegenerative syndrome combining Parkinsonism and NCLs [72–76]. ATP13A2 is a lysosomal transport protein that helps maintain optimal pH in lysosomes [46], and ceramide is metabolized in lysosomes [77]. The apoptosis that appears to cause NCLs is associated with increased levels of ceramide [78, 79], which have also been linked to *α*-synuclein deposition, which may contribute to PD pathogenesis [80]. It may be that ATP13A2 helps regulate ceramide metabolism, such that significant changes in ATP13A2 activity may contribute to the pathogenesis of both PD and NCLs. This model is similar to that of the lysosomal storage disorder called Gaucher disease. The homozygous mutations in the *β*-glucocerebrosidase gene that cause Gaucher disease also increase risk of PD [81]. Both diseases arise because lysosomal dysfunction leads to excessive aggregation of substrates that normally are degraded. Analogously, lysosomal dysfunction may underlie the clinically different neurodegenerative disorders of PD and NCLs.

## 6. Summary

Much has been learned about the physiological functions of ATP13A2 since mutations in the* ATP13A2* gene were first linked to autosomal recessive familial KRS [7]. Patients with such mutations show onset at earlier ages than patients with other forms of PD, as well as some atypical clinical symptoms such as pyramidal degeneration, supranuclear palsy, cognitive impairment, and dystonia. Studies in animal models of PD and in cultures of cells taken from patients with KRS and other types of PD suggest that ATP13A2 is important for proper functioning of lysosomes and mitochondria and perhaps for clearance of divalent metals; defects in any of these three processes are tightly associated with neurodegeneration. Nevertheless, more studies are needed that directly examine how PD-associated mutations in* ATP13A2* affect the activity and localization of the protein and ultimately the integrity of these three processes.* ATP13A2* mutations that affect one of these processes, lysosomal functioning, may simultaneously increase the risk of PD and NCLs. In other words, these quite clinically different diseases may share a mechanism of lysosomal dysfunction. If further studies validate the literature, the* ATP13A2 *gene and/or protein may become a suitable therapeutic target for treating both PD and NCLs.

## Figures and Tables

**Table 1:** Review of the literature on *ATP13A2* mutations associated with Parkinson's disease.

Ref.	Author	Year	Country of patient origin	Mutation	Notes
[7]	Ramirez et al.	2006	Chile, Jordan	c.3057delC (p.1019GfsX1021) c.1306+5G>A (p.G399_L435del) c.1632_1653dup22 (p.Leu552fsX788)	

[11]	Di Fonzo et al.	2007	Brazil, Italy	c.1510G>C (p.Gly504Arg) c.35C>T (p.Thr12Met) c.1597G>A (p.Gly533Arg)	

[12]	Ning et al.	2008	Japan	c.546C>A (p.Phe182Leu)	

[14]	Lin et al.	2008	Taiwan, Singapore	c.2236G>A (p.Ala746Thr)	Ethnic Chinese

[18]	Djarmati et al.	2009	Various European countries	c.746C>T (p.Ala249Val) c.844A>T (p.Ser282Cys) c.2939G>A (p.Arg980His)	
			Iran	c.1346G>A (p.Arg449Gln)	

[20]	Schneider et al.	2010	Pakistan	c.1103_1104insGA (p.Thr367fsX29)	

[25]	Fei et al.	2010	China (mainland)	c.2236G>A (p.Ala746Thr)	

[26]	Mao et al.	2010	China (mainland)	c.2236G>A (p.Ala746Thr)	

[13]	Funayama et al.	2010	Japan	c.2236G>A (p.Ala746Thr)	

[15]	Chen et al.	2011	Taiwan	c.3274A>G (p.Gly1014Ser)	Ethnic Chinese

[21]	Fong et al.	2011	Lithuania	c.1108_1120del13 (p.Arg370fsX390)	

[16]	Park et al.	2011	Various Asian countries	c.3176T>G (p.Leu1059Arg) c.3253delC (p.L1085wfsX1088)	

[22]	Crosiers et al.	2011	Afghanistan	c.2742_2743delTT (p.F851CfsX856)	

[23]	Eiberg et al.	2012	Greenland	c.2473C>AA (p.Leu825fs)	Ethnic Inuits

[17]	Zhu et al.	2012	China (mainland)	c.1754G>T (p. Ala585Asp)	Ethnic Chinese

[24]	Santoro et al.	2011	Italy	c.2629G>A (p.Gly877Arg)	

[27]	Chan et al.	2013	China (Hong Kong)	c.2236G>A (p.Ala746Thr)	Ethnic Chinese

[28]	Darvish et al.	2013	Iran	Deletion of exon 2	

[19]	Malakouti-Nejad et al.	2014	Iran	c.2762C>T (p.Gln858∗)	

**Table 2:** Clinical features of patients with Parkinson's disease and mutations in the *ATP13A2* gene.

Ref.	Internal code	Mutation	Country of origin	AO (years)	G	FH	IS	MS	MC	SUP	DYS	CD	H	BS	Response to levodopa	Imaging findings
[26]	V44	1632_1653dup22 (552LeufsX788)	Jordan	12	M	+	B, MR, R	B, R, PI	+	+	−	+	+	+	+	Diffuse atrophy (MRI)
	V48		Jordan	15	M	+	B, R	B, R, PI	+	+	+	+	+	+	+	Diffuse atrophy (MRI)
	V49		Jordan	13	M	+	MR, R	B, R, PI	+	−	+	+	+	+	+	Diffuse atrophy (MRI)
	V53		Jordan	12	F	+	B	B, R, PI	+	−	+	+	+	+	+	NR

[1]	II-8	c.3057delC (p.1019GfsX1021) c.1306+5G>A (p.G399_L435del)	Chile	18	M	+	BR, F	T, B, R	+	+	NR	+	+	+	Never tried	Enlarged sulci (CT)
	II-9		Chile	17	M	+	BR, B, R	T, B, R, PI	+	−	NR	+	+	−	−	Mild, diffuse atrophy; caudate hypointensity (MRI)
	II-10		Chile	15	F	+	B, F, BR	T, B, R, PI	+	+	NR	+	−	+	−	NR
	II-11		Chile	12	M	+	F, BR	T, B, R, PI	+	+	NR	+	+	+	−	Diffuse atrophy (CT)

[11]	BR-3042	c.1510G>C (Gly504Arg)	Brazil	12	M	−	B	B, R, PI	+	+	NR	+	+	+	+	Diffuse atrophy (CT)
	VE29	c.35C>T (Thr12Met)	Italy	30	M	+	NA	T, B, R, PI	NR	+	+	−	−	+	+	NR
	PK-69-1	c.1597C>A (Gly533Arg)	Italy	40	M	+	NA	B, R, PI	NR	+	+	−	+	+	+	NR

[18]	L-1349	c.746C>T (Ala249Val)	Germany	31	F	−	T	T, B, R, PI	NR	+	−	NR	+	−	+	Normal (MRI)
	L-1928	c.844A>T (Ser282Cys)	Norway	20	M	−	PI	R, PI	NR	+	−	NR	−	+	+	Normal (MRI)
	L-324	c.1346G>A (Arg449Gln)	Iran	36	M	−	T	B, R, PI	NR	+	−	NR	+	+	+	Cerebral atrophy (CT)
	P-55	c.2939G>A (Arg980His)	Serbia	35	F	−	PT	T, B, R, PI	NR	+	−	NR	+	+	+	Normal

[20]	NR	c.1103_1104insGA (p.Thr367fsX29)	Pakistan	16	M	−	B, MR	B, R, PI	−	+	+	+	+	+	+	Diffuse atrophy (MRI)

[22]	II-3	c.2742_2743delTT (p.F851CfsX856)	Afghanistan	10	M	−	B, MR	B, R, PI	+	+	+	+	−	+	+	Diffuse atrophy, bilateral hypointensity in putamina and caudate nuclei (MRI)

[21]	NR	c.1108_1120del13 (p.Arg370fsX390)	Lithuania	6	M	−	Dysarthria, DYS	T, B, R, PI	NR	−	+	−	NR	−	+	Normal (MRI)

[23]	VI-1	c.2473C>AA, (p.Leu825AsnfsX32)	Greenland	27	F	+	FA	NR	NR	NR	NR	+	+	+	NR	Diffuse atrophy (MRI)
	VI-6		Greenland	24	M	+	Weakness	NR	NR	NR	+	+	+	+	NR	Diffuse atrophy (MRI)
	V-1		Greenland	12	M	+	T	B, R	+	+	+	+	+	+	NR	Normal (MRI)
	V-3		Greenland	10	F	+	CD	T, B, R	−	−	−	−	+	−	NR	NR
	V-5		Greenland	29	F	+	GD	PI	+	+	−	+	−	+	NR	Diffuse atrophy (MRI)
	V-9		Greenland	15	F	+	B, MR	T, B, R	+	NR	NR	+	−	NR	NR	NR

[19]	X4015	c.2762C>T (p.Gln858∗)	Iran	14	F	+	Motor defect	T, B, R, PI	NR	+	+	+	−	+	+	Diffuse atrophy (MRI)
	X4041		Iran	10	M	+	B	T, B, R, PI	NR	+	+	+	−	+	+	Diffuse atrophy (MRI)
	R1042		Iran	30	M	+	NR	T, B, R, PI	NR	NR	NR	NR	NR	NR	+	NR

[24]	NAPO6	c.2629G>A (Gly877Arg)	Italy	10	M	−	GD	B, R, PI	+	+	+	+	−	+	+	Diffuse atrophy (MRI)

[12]	A	c.546C>A (Phe182Leu)	Japan	22	F	−	GD	T, B, R, PI	+	+	+	+	+	+	+	Diffuse atrophy (MRI)

[14]	F37	c.2236G>A (Ala746Thr)	China	53	F	−	NA	T, B, R	−	−	+	−	NR	+	+	Normal (MRI)
	EK1		China	50	M	−	NA	T, B, R, PI	−	−	+	−	NR	+	+	Normal (MRI)
	Y56		China	39	M	−	NA	T, B, R, PI	−	−	+	−	NR	+	+	Normal (MRI)

[15]	H1288	c.3274A>G (p.Gly1014Ser)	China	48	F	−	NA	T, B, R, PI	NR	NR	NR	NR	NR	NR	+	Normal (MRI)
	H496	c.2236G>A (Ala746Thr)	China	49	M	−	NA	T, B, R, PI	NR	NR	NR	NR	NR	NR	+	NR
	H2120	c.2236G>A (Ala746Thr)	China	51	F	−	NA	T, B, R, PI	NR	NR	NR	NR	NR	NR	+	NR

[16]	NR	c.3176C>G (Leu1059Arg), c.3253delC (L1085wfsX1088)	China	17	M	+	A	B, R	+	+	+	NR	−	+	+	Normal (MRI)
	NR		China	17	F	+	A, D	B, R	+	+	+	NR	−	+	+	Normal (MRI)

A: anxiety; AO: age of onset; B: bradykinesia; BS: Babinski sign; CD: cognitive dysfunction; D: depression; DYS: dystonia; F: female; FA: fatigue; FH: family history; G: gender; GD: gait disturbance; IS: initial symptom; M: male; MC: myoclonus; MS: motor symptom; NR: not reported; PI: postural instability; PT: postural tremor; R: rigidity; SUP: supranuclear upgaze palsy; T: tremor.

## References

[1] Lesage S, Brice A (2009). Parkinson's disease: from monogenic forms to genetic susceptibility factors. *Human Molecular Genetics*.

[2] Bonifati V (2014). Genetics of Parkinson's disease—state of the art. *Parkinsonism & Related Disorders*.

[3] Coppedè F (2012). Genetics and epigenetics of Parkinson's disease. *Scientific World Journal*.

[4] Lesage S, Lohmann E, Tison F, Durif F, Dürr A, Brice A (2008). Gene symbol: PARK2. Disease: parkinsonism, juvenile, autosomal recessive.. *Human genetics*.

[5] Bonifati V, Rizzu P, Van Baren MJ (2003). Mutations in the DJ-1 gene associated with autosomal recessive early-onset parkinsonism. *Science*.

[6] Li Y, Tomiyama H, Sato K (2005). Clinicogenetic study of PINK1 mutations in autosomal recessive early-onset parkinsonism. *Neurology*.

[7] Ramirez A, Heimbach A, Gründemann J (2006). Hereditary parkinsonism with dementia is caused by mutations in ATP13A2, encoding a lysosomal type 5 P-type ATPase. *Nature Genetics*.

[8] Tomiyama H, Yoshino H, Ogaki K (2011). PLA2G6 variant in Parkinson’s disease. *Journal of Human Genetics*.

[9] Di Fonzo A, Dekker MCJ, Montagna P (2009). FBXO7 mutations cause autosomal recessive, early-onset parkinsonian-pyramidal syndrome. *Neurology*.

[10] Bras J, Verloes A, Schneider SA, Mole SE, Guerreiro RJ (2012). Mutation of the parkinsonism gene ATP13A2 causes neuronal ceroid-lipofuscinosis. *Human Molecular Genetics*.

[11] Di Fonzo A, Chien HF, Socal M (2007). ATP13A2 missense mutations in juvenile Parkinsonism and young onset Parkinson disease. *Neurology*.

[12] Ning YP, Kanai K, Tomiyama H (2008). PARK9-linked parkinsonism in eastern Asia: mutation detection in ATP13A2 and clinical phenotype. *Neurology*.

[13] Funayama M, Tomiyama H, Wu RM (2010). Rapid screening of ATP13A2 variant with highresolution melting analysis. *Movement Disorders*.

[14] Lin CH, Tan EK, Chen ML (2008). Novel *ATP13A2* variant associated with Parkinson disease in Taiwan and Singapore. *Neurology*.

[15] Chen C-M, Lin C-H, Juan H-F (2011). ATP13A2 variability in Taiwanese Parkinson's disease. *American Journal of Medical Genetics B: Neuropsychiatric Genetics*.

[16] Park J, Mehta P, Cooper AA (2011). Pathogenic effects of novel mutations in the P-type ATPase ATP13A2 (PARK9) causing Kufor-Rakeb syndrome, a form of early-onset parkinsonism. *Human Mutation*.

[17] Zhu LH, Luo XG, Zhou YS (2012). Lack of association between three single nucleotide polymorphisms in the PARK9, PARK15, and BST1 genes and Parkinson's disease in the northern Han Chinese population. *Chinese Medical Journal*.

[18] Djarmati A, Hagenah J, Reetz K (2009). ATP13A2 variants in early-onset Parkinson's disease patients and controls. *Movement Disorders*.

[19] Malakouti-Nejad M, Shahidi GA, Rohani M (2014). Identification of p.Gln858^∗^ in ATP13A2 in two EOPD patients and presentation of their clinical features. *Neuroscience Letters*.

[20] Schneider SA, Paisan-Ruiz C, Quinn NP (2010). ATP13A2 mutations (PARK9) cause neurodegeneration with brain iron accumulation. *Movement Disorders*.

[21] Fong CY, Rolfs A, Schwarzbraun T, Klein C, O'Callaghan FJK (2011). Juvenile parkinsonism associated with heterozygous frameshift ATP13A2 gene mutation. *European Journal of Paediatric Neurology*.

[22] Crosiers D, Ceulemans B, Meeus B (2011). Juvenile dystonia-parkinsonism and dementia caused by a novel ATP13A2 frameshift mutation. *Parkinsonism and Related Disorders*.

[23] Eiberg H, Hansen L, Korbo L (2012). Novel mutation in ATP13A2 widens the spectrum of Kufor-Rakeb syndrome (PARK9). *Clinical Genetics*.

[24] Santoro L, Breedveld GJ, Manganelli F (2011). Novel *ATP13A2* (*PARK9*) homozygous mutation in a family with marked phenotype variability. *Neurogenetics*.

[25] Fei QZ, Cao L, Xiao Q (2010). Lack of association between ATP13A2 Ala746Thr variant and Parkinson’s disease in Han population of mainland China. *Neuroscience Letters*.

[26] Mao XY, Burgunder JM, Zhang ZJ (2010). ATP13A2 G2236A variant is rare in patients with early-onset Parkinson's disease and familial Parkinson's disease from mainland China. *Parkinsonism and Related Disorders*.

[27] Chan AYY, Baum L, Tang NLS (2013). The role of the Ala746Thr variant in the ATP13A2 gene among Chinese patients with Parkinson's disease. *Journal of Clinical Neuroscience*.

[28] Darvish H, Movafagh A, Omrani MD (2013). Detection of copy number changes in genes associated with Parkinson's disease in Iranian patients. *Neuroscience Letters*.

[29] Vilariño-Güell C, Soto AI, Lincoln SJ (2009). ATP13A2 variability in Parkinson disease. *Human Mutation*.

[30] Rakovic A, Stiller B, Djarmati A (2009). Genetic association study of the P-type ATPase ATP13A2 in late-onset Parkinson's disease. *Movement Disorders*.

[31] Najim Al-Din AS, Wriekat A, Mubaidin A, Dasouki M, Hiari M (1994). Pallido-pyramidal degeneration, supranuclear upgaze paresis and dementia: Kufor-Rakeb syndrome. *Acta Neurologica Scandinavica*.

[32] Williams DR, Hadeed A, Najim al-Din AS, Wreikat A, Lees AJ (2005). Kufor Rakeb disease: autosomal recessive, levodopa-responsive Parkinsonism with pyramidal degeneration, supranuclear gaze palsy, and dementia. *Movement Disorders*.

[33] Behrens MI, Brüggemann N, Chana P (2010). Clinical spectrum of Kufor-Rakeb syndrome in the Chilean kindred with ATP13A2 mutations. *Movement Disorders*.

[34] Schultheis PJ, Hagen TT, O’Toole KK (2004). Characterization of the P5 subfamily of P-type transport ATPases in mice. *Biochemical and Biophysical Research Communications*.

[35] Schröder B, Wrocklage C, Pan C (2007). Integral and associated lysosomal membrane proteins. *Traffic*.

[36] Matsui H, Sato F, Sato S (2013). ATP13A2 deficiency induces a decrease in cathepsin D activity, fingerprint-like inclusion body formation, and selective degeneration of dopaminergic neurons. *FEBS Letters*.

[37] Dehay B, Ramirez A, Martinez-Vicente M (2012). Loss of P-type ATPase ATP13A2/PARK9 function induces general lysosomal deficiency and leads to Parkinson disease neurodegeneration. *Proceedings of the National Academy of Sciences of the United States of America*.

[38] Murphy KE, Cottle L, Gysbers AM, Cooper AA, Halliday GM (2013). ATP13A2 (PARK9) protein levels are reduced in brain tissue of cases with Lewy bodies. *Acta Neuropathologica Communications*.

[39] Tofaris GK (2012). Lysosome-dependent pathways as a unifying theme in Parkinson's disease. *Movement Disorders*.

[40] Kong SM, Chan BK, Park JS (2014). Parkinson's disease-linked human PARK9/ ATP13A2 maintains zinc homeostasis and promotes α-Synuclein externalization via exosomes. *Human Molecular Genetics*.

[41] Gitler AD, Chesi A, Geddie ML (2009). α-Synuclein is part of a diverse and highly conserved interaction network that includes PARK9 and manganese toxicity. *Nature Genetics*.

[42] Usenovic M, Tresse E, Mazzulli JR, Taylor JP, Krainc D (2012). Deficiency of ATP13A2 leads to lysosomal dysfunction, α-synuclein accumulation, and neurotoxicity. *The Journal of Neuroscience*.

[43] Park JS, Koentjoro B, Veivers D, Mackay-Sim A, Sue CM (2014). Parkinson's disease-associated human ATP13A2 (PARK9) deficiency causes zinc dyshomeostasis andmitochondrial dysfunction. *Human Molecular Genetics*.

[44] Gusdon AM, Zhu J, van Houten B, Chu CT (2012). ATP13A2 regulates mitochondrial bioenergetics through macroautophagy. *Neurobiology of Disease*.

[45] Ramonet D, Podhajska A, Stafa K (2012). PARK9-associated ATP13A2 localizes to intracellular acidic vesicles and regulates cation homeostasis and neuronal integrity. *Human Molecular Genetics*.

[46] Nixon RA, Yang DS, Lee JH (2008). Neurodegenerative lysosomal disorders: a continuum from development to late age. *Autophagy*.

[47] Dehay B, Martinez-Vicente M, Caldwell GA (2013). Lysosomal impairment in Parkinson's disease. *Movement Disorders*.

[48] Olanow CW, Brundin P (2013). Parkinson's disease and alpha synuclein: is Parkinson's disease a prion-like disorder?. *Movement Disorders*.

[49] Bové J, Martínez-Vicente M, Vila M (2011). Fighting neurodegeneration with rapamycin: mechanistic insights. *Nature Reviews Neuroscience*.

[50] Schmidt K, Wolfe DM, Stiller B, Pearce DA (2009). Cd2+, Mn2+, Ni2+ and Se2+ toxicity to Saccharomyces cerevisiae lacking YPK9p the orthologue of human ATP13A2. *Biochemical and Biophysical Research Communications*.

[51] Mastroberardino PG, Hoffman EK, Horowitz MP (2009). A novel transferrin/TfR2-mediated mitochondrial iron transport system is disrupted in Parkinson’s disease. *Neurobiology of Disease*.

[52] Sheline CT, Zhu J, Zhang W, Shi C, Cai A (2013). Mitochondrial inhibitor models of Huntington's disease and Parkinson's disease induce zinc accumulation and are attenuated by inhibition of zinc neurotoxicity in vitro or in vivo. *Neurodegenerative Diseases*.

[53] Fukushima T, Tan X, Luo Y, Kanda H (2011). Serum vitamins and heavy metals in blood and urine, and the correlations among them in parkinson's disease patients in China. *Neuroepidemiology*.

[54] Hozumi I, Hasegawa T, Honda A (2011). Patterns of levels of biological metals in CSF differ among neurodegenerative diseases. *Journal of the Neurological Sciences*.

[55] Jiménez-Jiménez FJ, Fernández-Calle P, Martínez-Vanaclocha M (1992). Serum levels of zinc and copper in patients with Parkinson's disease. *Journal of the Neurological Sciences*.

[56] Guilarte TR (2010). Manganese and Parkinson's disease: a critical review and new findings. *Environmental Health Perspectives*.

[57] Pals P, van Everbroeck B, Grubben B (2003). Case-control study of environmental risk factors for Parkinson's disease in Belgium. *European Journal of Epidemiology*.

[58] Rentschler G, Covolo L, Ahmadi Haddad A, Lucchini RG, Zoni S, Broberg K (2012). ATP13A2 (PARK9) polymorphisms influence the neurotoxic effects of manganese. *NeuroToxicology*.

[59] Tan J, Zhang T, Jiang L (2011). Regulation of intracellular manganese homeostasis by Kufor-Rakeb syndrome-associated ATP13A2 protein. *Journal of Biological Chemistry*.

[60] Berg D, Hochstrasser H (2006). Iron metabolism in parkinsonian syndromes. *Movement Disorders*.

[61] Oakley AE, Collingwood JF, Dobson J (2007). Individual dopaminergic neurons show raised iron levels in Parkinson disease. *Neurology*.

[62] Devos D, Moreau C, Devedjian JC (2014). Targeting chelatable iron as a therapeutic modality in Parkinson’sdisease. *Antioxidants and Redox Signaling*.

[63] Poli M, Derosas M, Luscieti S (2010). Pantothenate kinase-2 (Pank2) silencing causes cell growth reduction, cell-specific ferroportin upregulation and iron deregulation. *Neurobiology of Disease*.

[64] Tsunemi T, Krainc D (2014). Zn^2+^ dyshomeostasis caused by loss of ATP13A2/PARK9 leads to lysosomal dysfunction and alpha-synuclein accumulation. *Human Molecular Genetics*.

[65] Xu J, Marzetti E, Seo AY, Kim J, Prolla TA, Leeuwenburgh C (2010). The emerging role of iron dyshomeostasis in the mitochondrial decay of aging. *Mechanisms of Ageing and Development*.

[66] Youdim MBH, Riederer P (1993). The role of iron in senescence of dopaminergic neurons in Parkinson's disease. *Journal of Neural Transmission, Supplement*.

[67] Karki P, Lee E, Aschner M (2013). Manganese neurotoxicity: a focus on glutamate transporters. *Annals of Occupational and Environmental Medicine*.

[68] Goebel HH, Gerhard L, Kominami E, Haltia M (1996). Neuronal ceroid-lipofuscinosis—late-infantile or Jansky-Bielschowsky type—revisited. *Brain Pathology*.

[69] Wöhlke A, Philipp U, Bock P (2011). A one base pair deletion in the canine ATP13A2 gene causes exon skipping and late-onset neuronal ceroid lipofuscinosis in the Tibetan terrier. *PLoS Genetics*.

[70] Farias FHG, Zeng R, Johnson GS (2011). A truncating mutation in ATP13A2 is responsible for adult-onset neuronal ceroid lipofuscinosis in Tibetan terriers. *Neurobiology of Disease*.

[71] Schultheis PJ, Fleming SM, Clippinger AK (2013). Atp13a2-deficient mice exhibit neuronal ceroid lipofuscinosis, limited α-synuclein accumulation and age-dependent sensorimotor deficits. *Human Molecular Genetics*.

[72] Burneo JG, Arnold T, Palmer CA, Kuzniecky RI, Oh SJ, Faught E (2003). Adult-onset neuronal ceroid lipofuscinosis (Kufs disease) with autosomal dominant inheritance in Alabama. *Epilepsia*.

[73] Berkovic SF, Carpenter S, Andermann F, Andermann E, Wolfe LS (1988). Kufs' disease: a critical reappraisal. *Brain*.

[74] Nijssen PCG, Brusse E, Leyten ACM, Martin JJ, Teepen JLJM, Roos RAC (2002). Autosomal dominant adult neuronal ceroid lipofuscinosis: parkinsonism due to both striatal and nigral dysfunction. *Movement Disorders*.

[75] Taschner PEM, de Vos N, Thompson AD (1995). Chromosome 16 microdeletion in a patient with juvenile neuronal ceroid lipofuscinosis (Batten disease). *The American Journal of Human Genetics*.

[76] Lavrov AY, Ilyna ES, Zakharova EY, Boukina AM, Tishkanina SV (2002). The first three Russian cases of classical, late-infantile, neuronal ceroid lipofuscinosis. *European Journal of Paediatric Neurology*.

[77] Bras J, Singleton A, Cookson MR, Hardy J (2008). Emerging pathways in genetic Parkinson's disease: potential role of ceramide metabolism in Lewy body disease. *FEBS Journal*.

[78] El Haddad S, Khoury M, Daoud M (2012). CLN5 and CLN8 protein association with ceramide synthase: biochemical and proteomic approaches. *Electrophoresis*.

[79] Persaud-Sawin D, Mousallem T, Wang C, Zucker A, Kominami E, Boustany RN (2007). Neuronal ceroid lipofuscinosis: a common pathway?. *Pediatric Research*.

[80] van Ham TJ, Thijssen KL, Breitling R, Hofstra RMW, Plasterk RHA, Nollen EAA (2008). *C. elegans* model identifies genetic modifiers of α-synuclein inclusion formation during aging. *PLoS Genetics*.

[81] Halperin A, Elstein D, Zimran A (2006). Increased incidence of Parkinson disease among relatives of patients with Gaucher disease. *Blood Cells, Molecules, and Diseases*.

